# Investigating the effect of sexual behaviour on oropharyngeal cancer risk: a methodological assessment of Mendelian randomization

**DOI:** 10.1186/s12916-022-02233-3

**Published:** 2022-01-31

**Authors:** Mark Gormley, Tom Dudding, Linda Kachuri, Kimberley Burrows, Amanda H. W. Chong, Richard M. Martin, Steven J. Thomas, Jessica Tyrrell, Andrew R. Ness, Paul Brennan, Marcus R. Munafò, Miranda Pring, Stefania Boccia, Andrew F. Olshan, Brenda Diergaarde, Rayjean J. Hung, Geoffrey Liu, Eloiza H. Tajara, Patricia Severino, Tatiana N. Toporcov, Martin Lacko, Tim Waterboer, Nicole Brenner, George Davey Smith, Emma E. Vincent, Rebecca C. Richmond

**Affiliations:** 1 MRC Integrative Epidemiology Unit, Population Health Sciences, Bristol Medical School, University of Bristol, Bristol, UK; 2 Bristol Dental Hospital and School, University of Bristol, Bristol, UK; 3 Department of Population Health Sciences, Bristol Medical School, University of Bristol, Bristol, UK; 4 Department of Epidemiology & Biostatistics, University of California San Francisco, San Francisco, USA; 5 University Hospitals Bristol and Weston NHS Foundation Trust National Institute for Health Research Bristol Biomedical Research Centre, University of Bristol, Bristol, UK; 6 University of Exeter Medical School, RILD Building, RD&E Hospital, Exeter, UK; 7 Genetic Epidemiology Group, World Health Organization, International Agency for Research on Cancer, Lyon, France; 8 School of Psychological Science, Faculty of Life Sciences, University of Bristol, Bristol, UK; 9 Section of Hygiene, University Department of Life Sciences and Public Health, Università Cattolica del Sacro Cuore, Roma, Italia; 10 Department of Woman and Child Health and Public Health - Public Health Area, Fondazione Policlinico Universitario A. Gemelli IRCCS, Roma, Italy; 11 Department of Epidemiology, Gillings School of Global Public Health, University of North Carolina, Chapel Hill, USA; 12 Department of Human Genetics, Graduate School of Public Health, University of Pittsburgh, and UPMC Hillman Cancer Center, Pittsburgh, USA; 13 Prosserman Centre for Population Health Research, Lunenfeld-Tanenbaum Research Institute, Sinai Health System, Toronto, Canada; 14 Dalla Lana School of Public Health, University of Toronto, Toronto, Canada; 15 Princess Margaret Cancer Centre, Toronto, Canada; 16 Department of Molecular Biology, School of Medicine of São José do Rio Preto, São Paulo, Brazil; 17 Albert Einstein Research and Education Institute, Hospital Israelita Albert Einstein, São Paulo, Brazil; 18 Department of Epidemiology, School of Public Health, University of São Paulo, São Paulo, Brazil; 19 Department of Otorhinolaryngology and Head and Neck Surgery, Research Institute GROW, Maastricht University Medical Center, Maastricht, The Netherlands; 20 Infections and Cancer Epidemiology, Deutsches Krebsforschungszentrum, Heidelberg, Germany; 21 School of Cellular and Molecular Medicine, University of Bristol, Bristol, UK

**Keywords:** Sexual behaviour, Oropharyngeal cancer, Head and neck cancer, Mendelian randomization

## Abstract

**Background:**

Human papilloma virus infection is known to influence oropharyngeal cancer (OPC) risk, likely via sexual transmission. However, sexual behaviour has been correlated with other risk factors including smoking and alcohol, meaning independent effects are difficult to establish. We aimed to evaluate the causal effect of sexual behaviour on the risk of OPC using Mendelian randomization (MR).

**Methods:**

Genetic variants robustly associated with age at first sex (AFS) and the number of sexual partners (NSP) were used to perform both univariable and multivariable MR analyses with summary data on 2641 OPC cases and 6585 controls, obtained from the largest available genome-wide association studies (GWAS). Given the potential for genetic pleiotropy, we performed a number of sensitivity analyses: (i) MR methods to account for horizontal pleiotropy, (ii) MR of sexual behaviours on positive (cervical cancer and seropositivity for *Chlamydia trachomatis*) and negative control outcomes (lung and oral cancer), (iii) Causal Analysis Using Summary Effect estimates (CAUSE), to account for correlated and uncorrelated horizontal pleiotropic effects, (iv) multivariable MR analysis to account for the effects of smoking, alcohol, risk tolerance and educational attainment.

**Results:**

In univariable MR, we found evidence supportive of an effect of both later AFS (IVW OR = 0.4, 95%CI (0.3, 0.7), per standard deviation (SD), *p* = < 0.001) and increasing NSP (IVW OR = 2.2, 95%CI (1.3, 3.8) per SD, *p* = < 0.001) on OPC risk. These effects were largely robust to sensitivity analyses accounting for horizontal pleiotropy. However, negative control analysis suggested potential violation of the core MR assumptions and subsequent CAUSE analysis implicated pleiotropy of the genetic instruments used to proxy sexual behaviours. Finally, there was some attenuation of the univariable MR results in the multivariable models (AFS IVW OR = 0.7, 95%CI (0.4, 1.2), *p* = 0.21; NSP IVW OR = 0.9, 95%CI (0.5 1.7), *p* = 0.76).

**Conclusions:**

Despite using genetic variants strongly related sexual behaviour traits in large-scale GWAS, we found evidence for correlated pleiotropy. This emphasizes a need for multivariable approaches and the triangulation of evidence when performing MR of complex behavioural traits.

**Supplementary Information:**

The online version contains supplementary material available at 10.1186/s12916-022-02233-3.

## Background

Head and neck squamous cell carcinoma (HNSCC) is a heterogeneous disease [1], which can originate from the mucosa of the oral cavity, oropharynx and larynx. Worldwide, there are over half a million incident cases each year, resulting in more than 200,000 deaths annually [2]. While using tobacco products and consuming alcohol are well-established risk factors across all HNSCC subsites, oral human papilloma virus (HPV) infection has been identified as another risk factor, particularly within the oropharyngeal subsite [3–6]. In developed countries such as the USA, 60–70% of oropharyngeal cancer (OPC) cases are reported to be HPV-positive [7], compared to only around 5% of all oral cancer (OC) cases. Oncogenic HPV type-16 (HPV16) is the most common type found in approximately 90% of HPV-positive oropharyngeal tumours [8–10]. Antibodies against HPV oncoproteins may be potential biomarkers for OPC, with case-control studies demonstrating an association with seropositivity for late (L1) and early (E1, E2, E4, E6, E7) HPV16 proteins [11–14].

HPV is thought to be sexually transmitted via oro-genital contact [9, 15–20] and may enter the oropharyngeal mucosa via abrasions in the reticulated tonsillar epithelium [21]. One large pooled analysis investigating the role of sexual behaviour in HNSCC showed an increased risk of OPC with having a history of six or more lifetime sexual partners (OR = 1.3, 95% confidence intervals (95%CI), (1.0, 1.5)) and four or more oral sex partners (OR = 2.3, 95%CI (1.4, 3.6)). A positive association was observed among men who had oral sex (OR = 1.6, 95%CI (1.1, 2.3)) and those with an earlier age at sexual debut (OR = 2.4, 95%CI (1.4, 5.1)) [15]. Conversely, there was no association reported between oral sex practice and head and neck cancer in a more recent meta-analysis of 17 studies (OR = 1.1, 95%CI (0.9, 1.4)), suggesting inconsistency in these findings, although 12 of these 17 studies failed to stratify by oral and oropharyngeal subsite [22]. Furthermore, associations have typically been investigated using case-control studies [5], with self-reported sexual behaviour which may be subject to recall bias and misreporting. Positive associations have also been found between sexual behaviour, sexually transmitted infections and other risk factors for HNSCC, such as smoking and alcohol consumption, indicating the possibility of confounding [23].

Mendelian randomization (MR) is an approach to causal analysis which attempts to overcome shortcomings of conventional observational studies by using single-nucleotide polymorphisms (SNPs) which are randomly allocated at conception and known to be reliably associated with modifiable risk factors of interest. These genetic instruments can be used to estimate the effects of risk factors on disease outcomes, in this case sexual behaviours on OPC [24, 25], which are less prone to unidentified confounding or reverse causation than conventional epidemiological analysis. Large-scale genome-wide association studies (GWAS) have been performed for sexual behaviour traits, including number of sexual partners (NSP) [26, 27] and age at first sex (AFS) [28], which will be the sexual behaviour outcomes investigated in this study. MR makes three key assumptions in that the genetic instrument (i) is robustly associated with the risk factor (i.e. ‘relevance’), (ii) does not share a common cause with the outcome (i.e. ‘exchangeability’) and (iii) affects the outcome only through the risk factor (i.e. ‘exclusion restriction principle’) to check for genetic pleiotropy [24, 25].

Here, we applied two-sample Mendelian randomization (MR) using summary-level genetic data from the largest available GWAS for each sexual behaviour (sample 1) and OPC (sample 2). We first conducted univariable MR analysis to assess the effects of NSP and AFS on OPC risk. We next performed univariable MR analysis to explore the effect of sexual behaviours on HPV seropositivity. Genetic proxies for complex human behaviours are more likely to have broad pleiotropic effects and may influence multiple upstream pathways that indirectly impact on sexual behaviour. In particular, genetic variants associated with sexual behaviour may also influence the disease outcome via other head and neck cancer risk factors, such as smoking and alcohol consumption. For this reason, we performed a number of sensitivity analyses: (i) MR methods to account for horizontal pleiotropy, (ii) MR of sexual behaviours on positive (cervical cancer and seropositivity for *Chlamydia trachomatis*) and negative control outcomes (lung and oral cancer), (iii) Causal Analysis Using Summary Effect estimates (CAUSE), to account for correlated and uncorrelated horizontal pleiotropic effects [29], (iv) multivariable MR analysis to account for the effects of smoking, alcohol, risk tolerance and educational attainment.

## Methods

### Summary-level data for sexual behaviours

Summary statistics for AFS were obtained from a GWAS conducted in the UK Biobank (*n* = 397,338) [30] [28]. AFS was treated as a continuous variable, with individuals considered as eligible if they had given a valid answer to the question “What was your age when you first had sexual intercourse? (Sexual intercourse includes vaginal, oral or anal intercourse)” and ages < 12 years old were excluded. Since AFS had a non-normal distribution, a within-sex inverse rank normal transformation was applied [28]. Where possible, the full 272 SNP AFS instrument was used, except in the primary analysis of OPC, whereby only 139 SNPs could be extracted from head and neck cancer data (Additional file 1). We obtained summary statistics for the NSP instrument (117 SNPs) from a GWAS conducted in UK Biobank [26] (*n* = 370,711) (Additional file 1). NSP was treated as a continuous variable based on responses to the question: “About how many sexual partners have you had in your lifetime?”. Respondents who reported > 99 lifetime sexual partners were asked to confirm their responses and a value of zero was assigned to participants who reported having never had sex, which was normalised separately for both males and females with an inverse rank normal transformation [26]. Both AFS and NSP GWAS adjusted for the top 10 principal components (accounting for population stratification), sex and birth year. For AFS, those participants with family data were controlled with non-independence of family members or else one family member was included in the analysis [28].

### Summary-level data for oropharyngeal cancer

The largest available GWAS for OPC was performed on 2641 OPC cases and 6585 matched controls from 12 studies which were part of the Genetic Associations and Mechanisms in Oncology (GAME-ON) Network [31]. Cancer cases comprised the following ICD-10 codes: oropharynx (C01.9, C02.4 and C09.0–C10.9). Stratification was conducted by geographical region to evaluate potential heterogeneity in any effects given potential differences in the distribution of genetic variants for specific traits within populations. As GAME-ON included participants from Europe (45.3%), North America (43.9%) and South America (10.8%), this study was restricted to individuals of predominantly European ancestry to avoid the effect of population structure. Details of the studies included as well as the genotyping and imputation performed have been described previously [31, 32].

### Univariable Mendelian randomization

To assess effects of NSP and AFS, we used SNPs which reached genome-wide significance (*p* < 5 × 10^-8^) and were determined to be independent in their respective GWAS [26, 28] using pairwise *r*^2^ < 0.1 (with 250-kb linkage disequilibrium (LD) windows). Further repeated analysis using a more stringent clumping threshold *r*^2^ < 0.001 was also conducted. Two-sample MR analyses were conducted using the “TwoSampleMR” package (version 0.5.5) in R (version 4.0.2) to extract the SNPs instrumenting the risk factor from the OPC GWAS. Harmonization of the direction of effects between exposure and outcome associations was performed, and palindromic SNPs were aligned when minor allele frequencies (MAFs) were less than 0.3 or were otherwise excluded. SNP-specific Wald estimates were calculated (SNP-outcome estimate divided by SNP-exposure estimate) and an inverse variance weighted (IVW) method applied to meta-analyse these in order to obtain an effect estimate of the risk factor on OPC risk.

### MR for sexual behaviours on HPV and *C. trachomatis* seropositivity

Where there was evidence for an effect of sexual behaviour on OPC risk, we also aimed to confirm the suspected aetiological link via HPV, by investigating the effects of NSP and AFS on a range of seropositivity measures against HPV16 L1 (*n* = 340 seropositive cases, *n* = 7566 controls), E6 (*n* = 126 seropositive cases, *n* = 7780 controls), E7 (*n* = 252 seropositive cases, *n* = 7654 controls) and HPV18 L1 (*n* = 191 seropositive cases, *n* = 7715 controls) proteins. Here, seropositivity suggests previous HPV exposure, which can be a predictor of cancer. Generally, HPV16 L1 antibodies are considered cumulative exposure markers, while HPV16 E6 and E7 have been associated with HPV-driven cancers but not all those who test positive are expected to develop a HPV-driven cancer [33]. Summary-level genetic data for HPV16 and HPV18 serological measures were obtained from UK Biobank. We performed individual GWAS for each measure using a similar approach as described by Kachuri et al. [34] using GWAS was performed using PLINK 2.0 (July 27, 2020, version) [35]. Details on how these GWAS were conducted can be found in Additional file 2: Supplementary information [12, 33, 36–40].

### Sensitivity analyses

The strength of each genetic instrument was determined by the magnitude and precision of association with the sexual behaviour, which was considered to be sufficient if the corresponding F-statistic was > 10. The fixed-effect IVW method provides an unbiased estimate in the absence of horizontal pleiotropy or when horizontal pleiotropy is balanced [41]. To account for directional pleiotropy, we compared results with three other MR methods, which each makes different assumptions about this: MR-Egger [42], weighted median [43] and weighted mode [44]. Scatter and leave-one-out plots were produced to evaluate influential outliers, and Mendelian Randomization Pleiotropy RESidual Sum and Outlier (MR-PRESSO) was applied to detect and correct for potential outliers (*p* < 0.05), using the MR-PRESSO package in R (version 4.0.2) [45]. Further detail on these methods is provided in Additional file 2: Supplementary information.

### Positive and negative control analyses

To further assess the specificity and sensitivity of the genetic instruments identified in relation to sexual behaviour, we conducted additional positive and negative control MR analyses. These were selected based on current evidence and aimed to appraise the role of AFS and NSP on (a) cervical cancer and *C. trachomatis* seropositivity, as positive control outcomes where evidence of an effect would support the aetiological link via HPV; and (b) lung cancer and oral cancer as negative controls, where a direct causal effect of sexual behaviour is unlikely and so where any evidence of an effect would indicate potential violation of the MR assumptions due to pleiotropy, population stratification or selection bias [46]. Details on the GWAS summary data used to conduct positive and negative control outcomes can be found in Additional file 2: Supplementary information [47, 48].

### Causal Analysis using Summary Effect estimates (CAUSE)

**Fig. 1:**
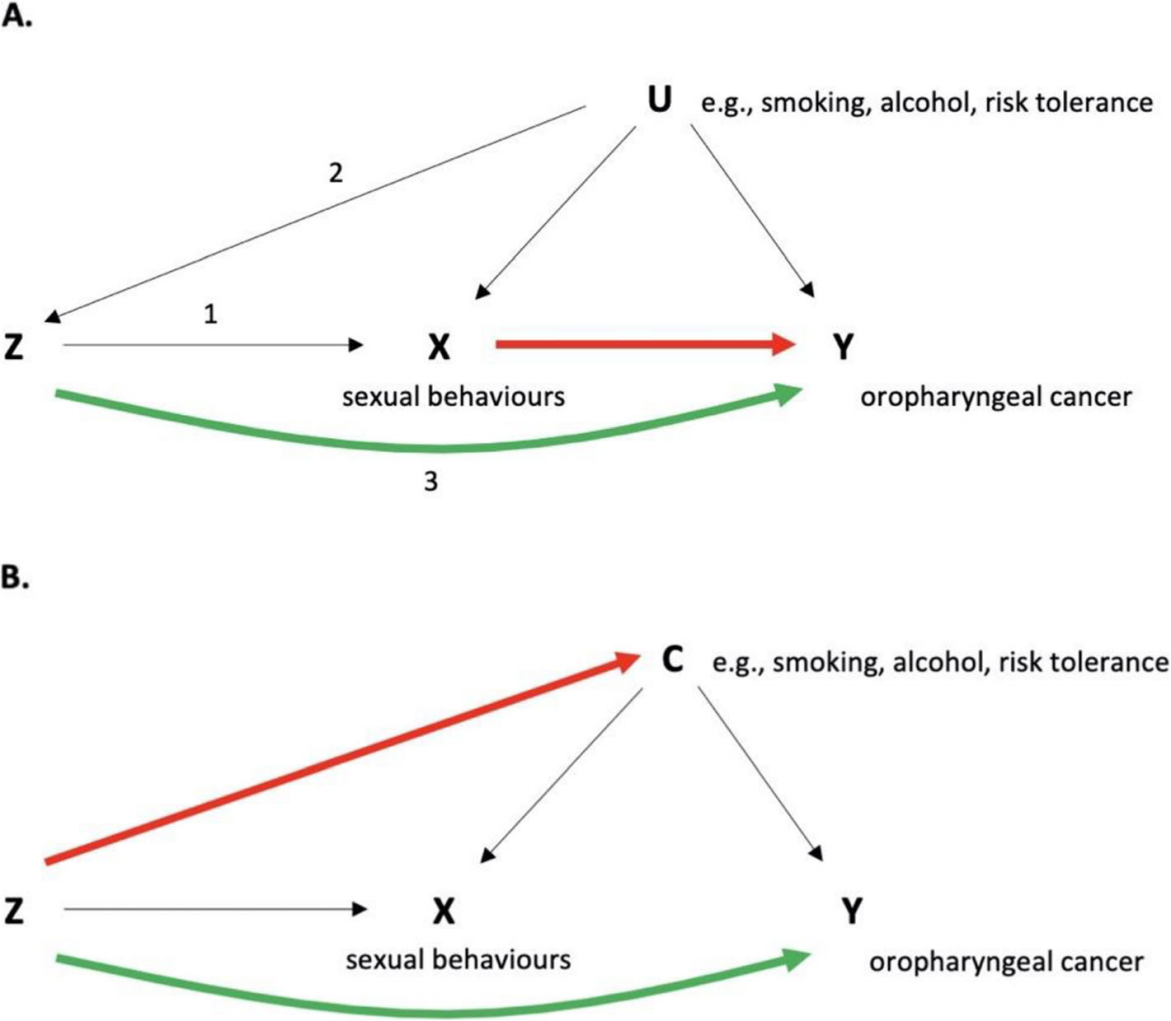
Directed acyclic graph (DAG) depicting Mendelian randomization and correlated pleiotropy. **A** Genetic variants (Z) act as proxies or instruments to investigate if an exposure (X) is associated with a disease outcome (Y). Causal inference can be made between X and Y if the following conditions are upheld: (1) Z is a valid instrument, reliably associated with X (‘relevance’); (2) Z is independent of any measured or unmeasured confounding factors (U) (‘exchangeability’) and (3) there is no independent association between Z and Y except through X (‘exclusion restriction’). **B** DAG depicting correlated pleiotropy (C) whereby the genetic variant (Z) can affect the exposure (X) and the outcome (Y) via a shared heritable factor (C), for example here through smoking, alcohol, or risk tolerance

While sensitivity analyses like MR-Egger, weighted median and weighted mode can detect horizontal or uncorrelated pleiotropy, whereby the genetic variant affects the exposure (sexual behaviours—AFS and NSP) and outcome (OPC) through separate mechanisms, correlated pleiotropy is an alternative scenario which could generate spurious associations in MR. Here, the genetic variant affects the exposure and outcome via a shared heritable factor. Correlated pleiotropy may be present in the genetic instruments for AFS and NSP, which if undetected could lead to false positive results (Fig. 1).

We used the CAUSE method in an attempt to identify potential correlated pleiotropy [29]. CAUSE proposes that any causal effect of an exposure on the outcome leads to correlation for all variants with a non-zero effect on the exposure, while a shared factor induces correlation for only a subset of exposure effect variants [29]. GWAS summary statistics were used to generate two models nested in a “null” effects model. The sharing model allows for horizontal pleiotropic effects but no causal effect (*γ* = 0), whereas the causal model has *γ* as a free parameter. The Bayesian expected log pointwise posterior density (ELPD) is used to compare models, producing a one-sided *p* value which tests the best fitting model. In particular, if the hypothesis that the sharing model fits the data at least as well as the causal model is rejected, we can conclude that the data are consistent with a causal effect [29].

### Multivariable Mendelian randomization

Genetic correlation was calculated between the two sexual behaviour traits (AFS and NSP), smoking, alcohol and risk tolerance using LD Score regression. Additionally, LD Score regression was conducted between AFS, NSP and HPV seropositivity. Further detail on this method can be found in Additional file 2: Supplementary information [49] [50]. To account for the potential genetic overlap with other risk factors [26] for OPC which may lead to correlated pleiotropy, we next conducted two-sample multivariable MR analysis. This accounted for the effects of the other sexual behaviour, smoking, alcohol consumption, risk tolerance and educational attainment in the MR of each sexual behaviour onto the cancer outcomes. First multivariable MR was carried out to assess the effect of genetic overlap between AFS and NSP using the genome-wide significant SNPs identified as instruments in the univariable analysis (272 SNPs for AFS and 117 SNPs for NSP). In total, 196 independent SNPs (*p* < 5 × 10^−8^) were used in the analysis for smoking initiation, 60 SNPs for alcoholic drinks per week [51], 123 for risk tolerance [26] and 317 SNPs for educational attainment after excluding SNPs with a pairwise *r*^2^ > 0.001 [52]. To better capture lifetime smoking (duration, heaviness and cessation), we used 108 SNPs which make up the comprehensive smoking index, derived by Wootton et al in the UK Biobank (*n* = 462,690) [53].

SNP overlap was assessed between all instruments. We used generalized versions of Cochran’s Q statistical tests for both instrument strength and validity [54]. Both the IVW and MR-Egger framework have been extended to estimate causal effects in multivariable MR analysis [55, 56], which was conducted using both the MVMR (version 0.2.0) and MendelianRandomization [57] (version 0.5.0) packages in R (version 4.0.2). To further clarify the direction of causal effect between AFS, NSP and other risk factors (including smoking initiation, the comprehensive smoking index, alcohol drinks per week, risk tolerance and educational attainment), bidirectional MR was conducted.

Causal Analysis using Summary Effect Estimates, LD Score Regression and multivariable Mendelian randomization approaches all require full GWAS summary data for the proposed risk factors of interested. Full data were available for the GWAS of NSP [26], but these have yet to be published for the GWAS of AFS. Therefore, for these approaches, we used another GWAS for AFS, also conducted using UK Biobank data (*n* = 406,457), for which full summary data are publicly available (https://gwas.mrcieu.ac.uk/datasets/ukb-b-6591/). This GWAS was conducted using the MRC IEU UK Biobank GWAS pipeline, more details of which can be found in Elsworth et al. [58].

## Results

### Univariable Mendelian Randomization

Using 139 SNPs robustly and independently associated with AFS (Additional file 1), there was evidence of a protective effect of later AFS on OPC (IVW OR = 0.4, 95%CI (0.3, 0.7), per standard deviation (SD), *p* = < 0.001) which was consistent across methods robust to horizontal pleiotropy (MR-Egger, weighted median and weighted mode) (Table 1 & Additional file 2: Fig. S1A). Using 117 SNPs (Additional file 1) independently associated with NSP, we found evidence to suggest an adverse effect of increased NSP on the risk of OPC (IVW OR = 2.2, 95%CI (1.3, 3.8) per SD, *p* = < 0.001). These results were consistent across the other MR methods (Table 1 & Additional file 2: Fig. S1B). Using a more stringent clumping threshold *r*^2^ < 0.001, the results for both AFS and NSP were comparable with the main analysis are included in Additional file 2: Table S1. The protective effect of later AFS was consistent across all geographical regions, with the most precise effects seen in the European (IVW OR = 0.4, 95%CI (0.2, 0.8), *p* = < 0.001) and North American population (IVW OR = 0.4, 95%CI (0.2, 0.8), *p* = 0.01) (Table 2). There was also suggestive evidence for an adverse effect of increasing NSP across regions, with the strongest effect again in the North American population (IVW OR = 3.0, 95%CI (1.4, 6.5), *p* = 0.01) (Table 2).

**Table 1:** Univariable Mendelian randomization results for age at first sex and number of sexual partners on risk of oropharyngeal cancer

Outcome	Exposure/ Outcome datasets	Outcome *N*	Controls *N*	Method	Age at first sex (*N* SNPs 139)		Number of sexual partners (*N* SNPs 117)	
					OR (95%CI)	***P***	OR (95%CI)	***P***
OPC	UK Biobank/ GAME-ON	2641	6585	IVW	0.44 (0.28, 0.70)	< 0.001	2.20 (1.27, 3.81)	< 0.001
OPC	UK Biobank/ GAME-ON	2641	6585	Weighted median	0.41 (0.23, 0.75)	< 0.001	2.57 (1.24, 5.29)	0.01
OPC	UK Biobank/ GAME-ON	2641	6585	Weighted mode	0.23 (0.04, 1.34)	0.10	3.57 (0.58, 21.69)	0.17
OPC	UK Biobank/ GAME-ON	2641	6585	MR-Egger	0.21 (0.03, 1.37)	0.10	1.88 (0.12, 29.49)	0.65

Abbreviations: *OPC*, oropharyngeal cancer; *IVW*, inverse variance weighted; *OR*, odds ratio; *CI*, confidence intervals; *P*, *p* value; *NSP*, number of sexual partners; *AFS*, age at first sex. AFS OR represents the exponential change in odds of oropharyngeal squamous cell carcinoma per SD change (7.3-month delay) in age at first sex. NSP OR represents the exponential change in odds of oropharyngeal squamous cell carcinoma per SD increase (0.94) in the number of sexual partners

**Table 2:** Inverse variance weighted univariable Mendelian randomization results for age at first sex and number of sexual partners on risk of oropharyngeal cancer, by region

Outcome	Region	N SNPs	Outcome *N*	Control *N*	Method	OR	CIL	CIU	*P* value
Age at first sex									
Oropharyngeal cancer	Europe	139	1090	2928	IVW	0.36	0.17	0.78	< 0.001
Oropharyngeal cancer	North America	139	1119	2329	IVW	0.41	0.20	0.83	0.01
Oropharyngeal cancer	South America	139	205	727	IVW	0.38	0.07	1.95	0.24
Number of sexual partners									
Oropharyngeal cancer	Europe	117	1090	2928	IVW	1.48	0.66	3.33	0.35
Oropharyngeal cancer	North America	117	1119	2329	IVW	2.99	1.37	6.51	0.01
Oropharyngeal cancer	South America	117	205	727	IVW	2.68	0.56	12.75	0.22

Abbreviations: *SNPs*, single-nucleotide polymorphisms; *IVW*, Inverse variance weighted; *SE*, standard error; *OR*, odds ratio; *CIL*, lower confidence interval; *CIU*, upper confidence interval; *P p* value. OR represents the exponential change in odds of oropharyngeal squamous cell carcinoma per SD change (7.3-month delay) in age at first sex/ or per SD increase (0.94) in number of sexual partners

### MR for effect of sexual behaviours on HPV seropositivity

Using the NSP and AFS instruments, we next evaluated the effect of sexual behaviour on the risk of HPV seropositivity in healthy individuals, using a GWAS of serological measures in UK Biobank. There appeared to be some evidence for a protective effect of later AFS (IVW OR = 0.5, 95%CI (0.2, 1.0), *p* = 0.05) on HPV16 L1 seropositivity (Additional file 2: Table S2). However, there was limited evidence for a similar protective effect on HPV18 L1, HPV16 E6 or E7 seropositivity. While there was some evidence that increasing NSP also increased the likelihood of HPV16 E6 seropositivity (IVW OR = 5.4, 95%CI (1.0, 28.3), *p* = 0.05), this was inconsistent among the other tested HPV antibodies (Additional file 2: Table S3).

### Sensitivity analyses

There was limited evidence of weak instrument bias (F-statistic > 10) and the proportion of variance in the phenotype (*R*^2^) explained by the genetic instruments ranged from 1 to 2% (Additional file 2: Table S4). There was limited evidence for heterogeneity in the SNP effect estimates for the AFS instrument (QIVW 159.4, *p =* 0.10; Q MR-Egger 158.6, *p* = 0.10), but clear evidence of heterogeneity in the NSP instrument (QIVW 155.6, *p =* 0.007; Q MR-Egger 155.6, *p* = 0.006) (Additional file 2: Table S5).

MR-Egger intercepts were not indicative of directional pleiotropy (Additional file 2: Table S5), but there were outliers present on visual inspection in both scatter and leave-one-out plots (Additional file 2: Fig. S2 & S3). MR-PRESSO identified 8 outliers for AFS and 7 outliers for NSP, which when corrected for, yielded effects consistent with univariable MR for both instruments (Additional file 2: Tables S6-8). There was evidence of violation of the NOME assumption for both AFS and NSP genetic instruments (i.e. *I*^2^ statistic < 0.90) (Additional file 2: Table S9), so MR-Egger was performed with SIMEX correction. The effects were consistent with previous MR-Egger results for AFS, but there was attenuation of the NSP effect on OPC (SIMEX corrected MR-Egger OR = 3.6, 95%CI (0.4, 32.1), *p* = 0.25) (Additional file 2: Table S9). These estimates should however be interpreted with caution, given evidence of high dilution in the SNP-exposure effects [59].

### Positive and negative control analyses

Univariable MR analysis conducted within UK Biobank found a protective effect for later AFS on cervical cancer, which is known to be another HPV-driven cancer type (IVW OR = 0.4, 95%CI (0.3, 0.7), *p* = < 0.001) (Additional file 2: Table S10). A similar effect was found when assessing the effect of AFS on *C. trachomatis* seropositivity based on pGP3 antigen, another positive control (IVW OR = 0.4, 95%CI (0.3, 0.6), *p* = < 0.001) (Additional file 2: Table S10). There was also evidence for an adverse effect of increasing NSP on cervical cancer risk (IVW OR = 1.9, 95CI% (1.0, 3.9), *p* = 0.06) and a positive association between NSP and *C. trachomatis* serostatus (IVW OR = 2.4, 95%CI (1.4, 4.1), *p* = < 0.001) (Additional file 2: Table S11).

Using lung cancer as a negative control, in univariable MR there was a strong protective effect of AFS (IVW OR = 0.1 95%CI (0.1, 0.3), *p* = < 0.001) (Additional file 2: Table S10) and an adverse effect of increasing NSP (IVW OR = 7.1 95%CI (2.4, 21.6), *p* = < 0.001) (Additional file 2: Table S11), indicating violation of the MR assumptions. A protective effect was also observed in relation to AFS with oral cancer, another negative control (IVW OR = 0.6, 95%CI (0.4, 1.0), *p* = 0.03) (Additional file 2: Table S10); however, there was no effect for NSP on oral cancer (IVW OR = 1.2, 95%CI (0.7, 2.0), *p* = 0.47) (Additional file 2: Table S11).

While there was no strong evidence for directional pleiotropy (Additional file 2: Table S12), there was some evidence of heterogeneity (Additional file 2: Table S13) for both AFS and NSP in the lung and oral cancer analyses, suggesting that pleiotropy may be present [41]. While scatter and leave-one-out plots showed no obvious outliers (Additional file 2: Fig. S4-7), MR-PRESSO identified outliers for AFS and for NSP across all positive and negative controls. When corrected for outliers, the lung cancer results remained consistent with the univariable MR, suggesting further violation of the MR assumptions for the AFS and NSP instruments even after accounting for the outliers (Additional file 2: Table S14-15).

### Investigating correlated pleiotropy using CAUSE

We used GWAS summary statistics to evaluate evidence for an effect of AFS and NSP on OPC, using the Causal Analysis using Summary Effect estimates (CAUSE) method to account for correlated pleiotropy [60]. For AFS, CAUSE suggested there was relatively similar evidence for sharing (correlated pleiotropy) (*p* = 0.02) and causal models (*p* = 0.05) compared to the null (no effect) model (Additional file 2: Table S16 & Additional file 2: Fig. S8). Comparing both shared and causal models, there was limited evidence that the causal model fit the data better than the sharing model (*p =* 0.44), indicating that correlated pleiotropy could not be discounted. When investigating the causal effect of NSP on OPC, neither shared (*p* = 0.30) nor causal (*p* = 0.27) models appeared to fit in comparison to the null model, providing limited evidence for a causal effect of NSP (Additional file 2: Table S17 & Additional file 2: Fig. S9).

### Multivariable Mendelian randomization

In total there were 21 overlapping SNPs identified between genetic instruments (Additional file 2: Table S18) and LD score regression highlighted strong genetic correlation between the exposure traits (*rg* = |0.62–0.64|) (Additional file 2: Table S19 & Additional file 2: Fig. S10). A weak correlation was observed between AFS and HPV seropositivity (*rg* = |0.04–0.09|) as well as between NSP and HPV seropositivity (*rg* = |0.07–0.15|) (Additional file 2: Fig. S11).

**Fig. 2:**
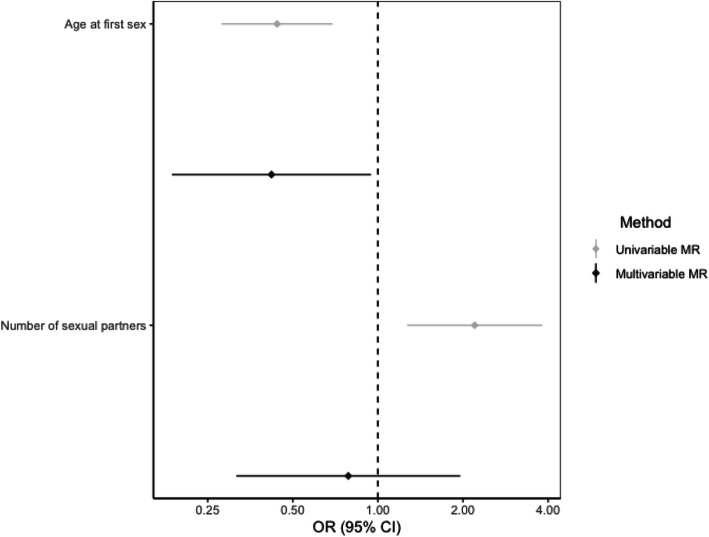
Forest plot comparing univariable and multivariable Mendelian randomization effects of age at first sex and number of sexual partners on oropharyngeal cancer risk. Effect estimates are reported on the log odds scale with 95% confidence intervals. Age at first sex point estimate represents the exponential change in odds of oropharyngeal squamous cell carcinoma per SD change (7.3-month delay) in age at first sex. Number of sexual partners point estimate represents the exponential change in odds of oropharyngeal squamous cell carcinoma per SD increase (0.94) in the number of sexual partners

**Fig. 3:**
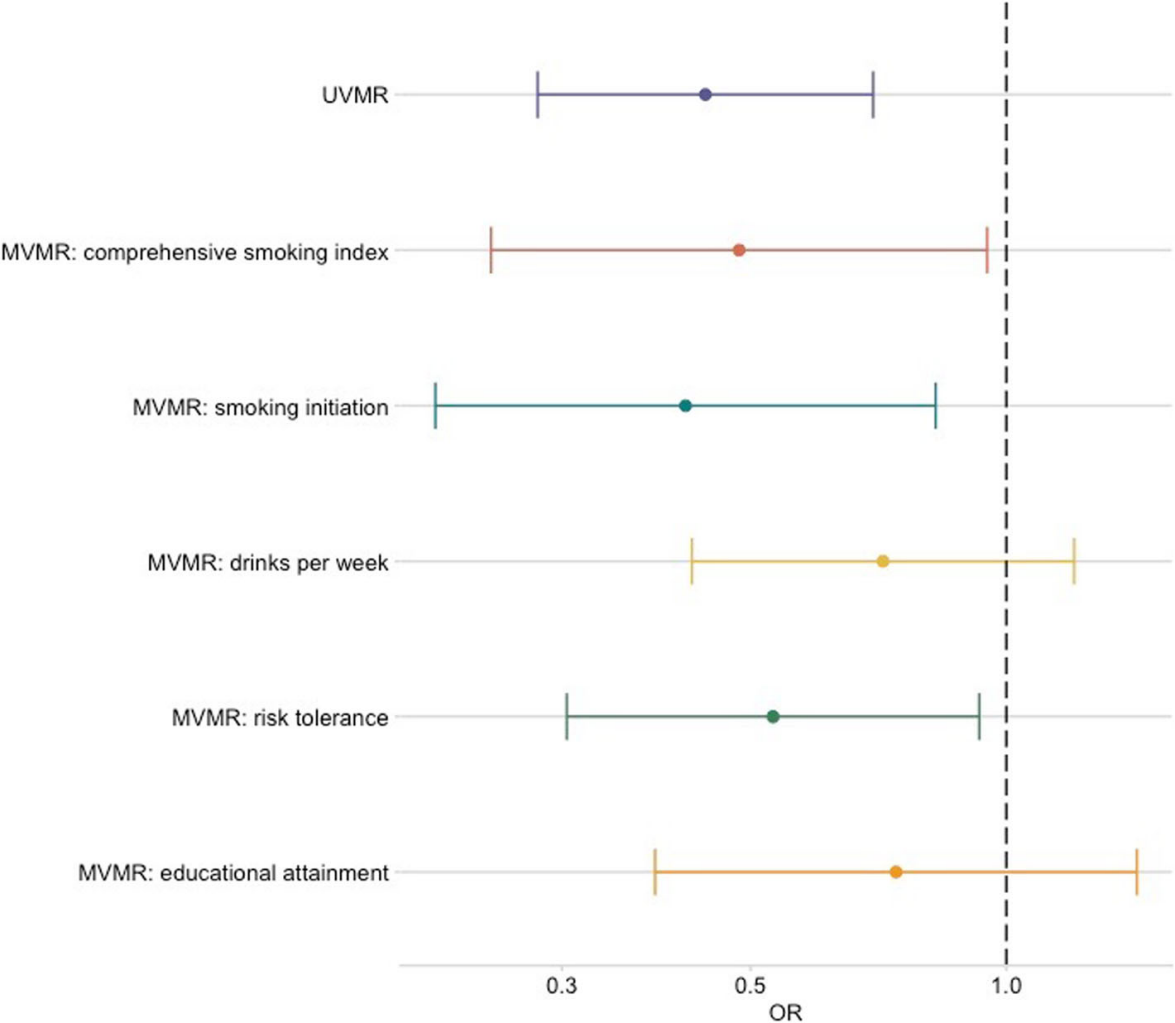
Forest plot showing multivariable Mendelian randomization results for age at first sex with risk of oropharyngeal cancer. Effect estimates on oropharyngeal cancer risk are reported on the log odds scale with 95% confidence intervals. UVMR, univariable Mendelian randomization; MVMR, multivariable Mendelian randomization; Age at first sex OR represents the change in odds of oropharyngeal squamous cell carcinoma per SD change (7.3-month delay) in age at first sex. Comprehensive smoking index (dark orange), smoking initiation (teal blue), alcoholic drinks per week (yellow), risk tolerance (green), educational attainment (light orange). The MVMR effect is the MR effect after accounting for this variable

**Fig. 4:**
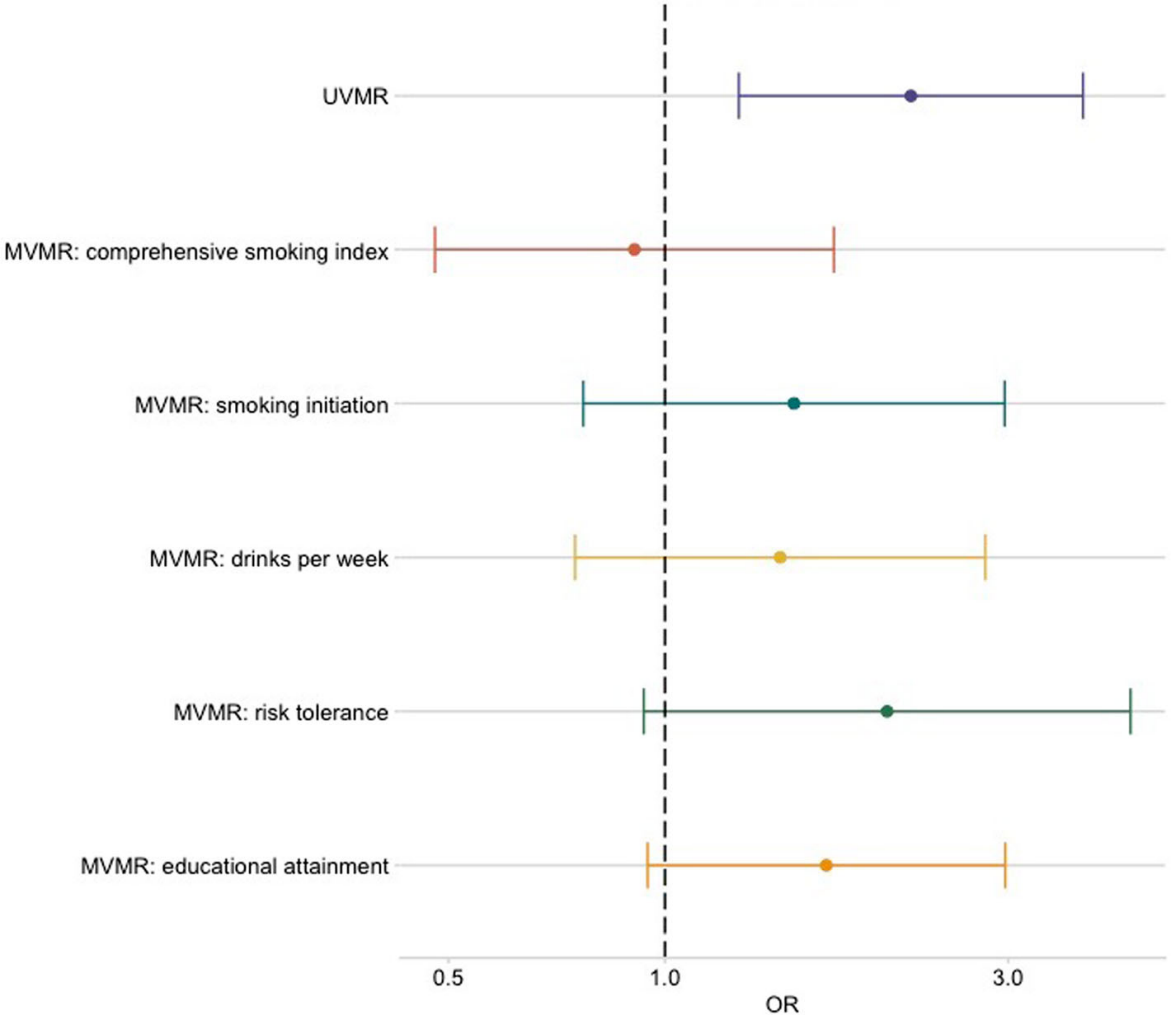
Forest plot showing multivariable Mendelian randomization results for number of sexual partners with risk of oropharyngeal cancer. Effect estimates on oropharyngeal cancer risk are reported on the log odds scale with 95% confidence intervals. UVMR, univariable Mendelian randomization; MVMR, multivariable Mendelian randomization; Number of sexual partners OR represents the change in odds of oropharyngeal squamous cell carcinoma per SD change (0.94) in number of sexual partners. Comprehensive smoking index (dark orange), smoking initiation (teal blue), alcoholic drinks per week (yellow), risk tolerance (green), educational attainment (light orange). The MVMR effect is the MR effect after accounting for this variable

Multivariable MR analysis was therefore carried out to investigate the direct causal effect of AFS and NSP on OPC after accounting for the other sexual behaviour, smoking, alcohol and risk tolerance. While the effect of NSP diminished (IVW OR = 0.8, 95%CI (0.3, 2.0), *p* = 0.60), the AFS effect remained (IVW OR = 0.4, 95%CI (0.2, 0.9), *p* = 0.04), after accounting for the other sexual behaviour in multivariable MR (Tables 3 and 4; Fig. 2). When accounting for smoking and risk tolerance, the effect of AFS remained consistent within the oropharyngeal subsite (Table 3 and Fig. 3). However, there was attenuation of the effect for AFS towards the null when controlling for drinks per week (IVW OR = 0.7, 95%CI (0.4, 1.2), *p* = 0.21) and educational attainment (IVW OR= 0.7, 95%CI (0.4, 1.4), *p* = 0.37). There was also some attenuation towards the null when investigating the effect of NSP on OPC accounting for lifetime smoking (IVW OR = 0.9, 95%CI (0.5 1.72), *p* = 0.76), alcohol consumption (IVW OR = 1.5, 95%CI (0.8, 2.8), *p* = 0.27), risk tolerance (IVW OR = 2.0, 95%CI (0.9, 4.4), *p* = 0.07) and educational attainment (IVW OR = 1.7, 95%CI (1.0, 3.0), *p* = 0.07) (Table 4 and Fig. 4).

**Table 3:** Multivariable Mendelian randomization for age at first sex with risk of oropharyngeal cancer

Exposure	Exposure dataset	N SNPs	Conditional F-stat	Q-stat	*P* value for instrument validity	Method	AFS OR	95% CI	*P*
Number of sexual partners	UK Biobank [26]	152	7.81	214.14	3.77 × 10^-4^	IVW	0.42	0.19, 0.94	0.04
						MR-Egger	0.24	0.06, 1.01	0.05
Comprehensive Smoking Index	UK Biobank [53]	174	8.87	191.26	0.14	IVW	0.48	0.25, 0.95	0.03
						MR-Egger	0.71	0.23, 2.19	0.56
Smoking initiation	GSCAN [51]	215	6.43	250.58	0.04	IVW	0.42	0.21, 0.83	0.01
						MR-Egger	0.61	0.21, 1.74	0.35
Drinks per week	GSCAN [51]	147	28.88	164.77	0.11	IVW	0.72	0.43, 1.20	0.21
						MR-Egger	0.43	0.14	1.28
Risk tolerance	UK Biobank [26]	160	13.68	171.18	0.21	IVW	0.53	0.30, 0.93	0.03
						MR-Egger	0.24	0.08, 0.72	0.01
Educational attainment	SSAGC and UK Biobank [52]	317	7.91	319.62	0.28	IVW	0.74	0.39, 1.43	0.37
						MR-Egger	0.79	0.33, 1.86	0.59

Abbreviations: *IVW*, inverse variance weighted; *AFS*, age at first sex; *OR*, odds ratio; *CI*, confidence intervals; *P p* value; *Q-stat*, Cochran’s Q statistic; *F-stat*, conditional F-statistic; Social Science Genetic Association Consortium (SSGAC). AFS OR represents the odds ratio of oropharyngeal squamous cell carcinoma per SD change (7.3-month delay) in age at first sex

**Table 4:** Multivariable Mendelian randomization for number of sexual partners with risk of oropharyngeal cancer

Exposure	Exposure dataset	N SNPs	Conditional F-stat	Q-stat	*P* value for instrument validity	Method	NSP OR	95% CI	*P*
Age at first sex	UK Biobank [28]	152	7.26	214.14	3.77 × 10^-4^	IVW	0.79	0.32, 1.96	0.60
						MR-Egger	0.97	0.35, 2.65	0.95
Comprehensive Smoking Index	UK Biobank [53]	157	12.18	168.74	0.20	IVW	0.91	0.48, 1.72	0.76
						MR-Egger	0.86	0.39, 1.88	0.70
Smoking initiation	GSCAN [51]	195	7.35	204.67	0.25	IVW	1.51	0.77, 2.97	0.23
						MR-Egger	1.66	0.66, 4.15	0.28
Drinks per week	GSCAN [51]	117	22.7	151.65	0.011	IVW	1.45	0.75, 2.79	0.27
						MR-Egger	1.61	0.76, 3.41	0.21
Risk tolerance	UK Biobank [26]	125	7.06	145.56	0.072	IVW	2.04	0.93, 4.44	0.07
						MR-Egger	2.12	0.66, 6.83	0.21
Educational attainment	SSAGC and UK Biobank [52]	317	42.37	326.36	0.28	IVW	1.67	0.95, 2.97	0.07
						MR-Egger	1.20	0.51, 2.83	0.67

Abbreviations: *IVW*, inverse variance weighted; *NSP*, number of sexual partners; *OR*, odds ratio; *CI*, confidence intervals; *P p* value; *Q-stat*, Cochran’s Q statistic; *F-stat*, conditional F-statistic; Social Science Genetic Association Consortium (SSGAC). NSP OR represents the odds ratio of oropharyngeal squamous cell carcinoma per SD increase (0.94) in the number of sexual partners

These results suggest the NSP and AFS instruments may include pleiotropic variants related to smoking and drinking behaviours. Some of the multivariable models including smoking initiation and drinks per week showed high levels of heterogeneity and therefore further risk of invalid instruments (Tables 3 and 4). However, the MR-Egger intercepts in the multivariable analyses were consistent with the null, indicative of no further directional pleiotropy (Additional file 2: Table S20) and the effects estimated were also consistent across both IVW and MR-Egger models (Tables 3 and 4). Additionally, with the exception of risk tolerance, there was a consistent bidirectional relationship between AFS and other risk factors (including the comprehensive smoking index, smoking initiation, alcohol drinks per week), and conversely a positive relationship between these risk factors and NSP using bidirectional MR. Similarly, increased educational attainment increased later age at first sex and results in decreased numbers of sexual partners. This indicates that the comprehensive smoking index, smoking initiation, alcohol drinks per week and educational attainment may serve as both confounders and mediators. However, this will be accounted for in the multivariable MR analysis, which provides a direct estimate of the effect for AFS and NSP (Additional file 2: Tables S21 & S22).

In additional multivariable MR analysis of AFS and NSP on lung cancer, effects for both instruments were attenuated once smoking was included in the model. With AFS, this was clearly seen when controlling for smoking initiation (IVW OR = 1.1, 95%CI (0.8, 1.6), *p* = 0.57) and a change in direction of the effect of AFS was evident when controlling for the comprehensive smoking index (IVW OR = 2.0, 95%CI (1.3, 3.0), *p* < 0.001) (Additional file 2: Table S23 & Additional file 2: Fig. S12). Similarly, there was limited evidence for an effect of NSP on lung cancer when controlling for the comprehensive smoking index (IVW OR = 0.7, 95%CI (0.4, 1.1), *p* = 0.09). The MR-Egger intercept deviated from the null in the multivariable models including smoking, suggestive of further directional pleiotropy in this analysis (Additional file 2: Table S24).

## Discussion

In this study, we applied Mendelian randomization to evaluate the effects of both later age at first sex and increased number of sexual partners on the risk of OPC. We observed convergence between genetic pathways influencing sexual behaviours and susceptibility to OPC, which may be partly mediated by HPV infection, however, we also uncovered complex correlated pleiotropy with other putative risk factors. Univariable MR results suggested a protective effect of later age at first sex and an adverse effect of increased number of sexual partners. However, these effects attenuated in the multivariable MR analyses that controlled for smoking behaviour and alcohol consumption. Adjusting for educational attainment appears to play an important role in the multivariable MR analysis for AFS, but less so for NSP, whereby the comprehensive smoking index resulted in the largest attenuation of the effect.

While there was suggestive evidence for an effect of sexual behaviours on some HPV16 serology measures and in cervical cancer (supportive of a causal mechanism via HPV infection), the same direction of effect was observed in negative control analysis (lung and oral cancer) indicating potential violation of the MR assumptions. Furthermore, CAUSE provided less support for a causal effect of AFS and NSP on OPC risk, highlighting the risk of correlated pleiotropy in the genetic instruments for these complex behavioural traits.

### Sexual behaviours and HPV transmission

Over 90% of HPV-positive OPC is caused by the high-risk genotype 16, with almost all oral infections thought to be sexually acquired [61]. HPV is a small non-enveloped DNA virus, with its genome encoding for both early oncoproteins E6/E7 and the late capsid proteins such as L1. The overexpression of these oncogenes is thought to stimulate proliferation and lateral expansion of epithelial basal cells, progressing to a malignant phenotype. HPV E6 forms a complex which leads to rapid degradation of tumour suppressor protein p53, resulting in deregulation of cell cycle checkpoints. E7 binds to a complex which ubiquitinates another tumour suppressor protein, retinoblastoma (pRb), again resulting in uncontrolled G1/S phase of the cell cycle [62]. While the transmission of HPV via sexual intercourse is well known and HPV, in turn, is a major risk factor for cervical malignancies, the role of HPV in OPC risk has only been acknowledged in recent decades [8]. Among OPC cases, HPV16 E6 serology is a good biomarker (~ 99% specificity, > 90% sensitivity) and therefore both E6 and E7 are highly associated with this disease [33]. However, when studying these antibodies in the general population, E6 seroprevalence appears to be very low (0.5–1%), but in comparison with low incidence rates of HPV-positive OPC, this figure is still high, suggesting that not all individuals in the general population who have HPV16 E6 seropositivity will develop an oropharyngeal tumour or other HPV-associated cancer [33]. Consequently, we performed this analysis in UK Biobank and observed a strong and consistent association with sexual behaviour. In our univariable MR analysis, the effects of AFS and NSP instruments on risk of HPV16 and HPV18 seropositivity were not consistent, compared with recent observational studies which demonstrate an association between serology markers and sexual behaviour responses in UK Biobank [33]. This could be as a result of the small number of seropositive HPV16 (*n* = < 450) and HPV18 (*n* = 265) cases within the UK Biobank pilot study used in our genetic analysis or that results from genetic proxies and questionnaire data are not directly comparable [63]. Using serology measures to predict HPV seropositivity or a HPV-positive OPC diagnosis is not straightforward, often requiring the use of multiple markers simultaneously [64]. Going forward, more reliable tests may emerge which could improve our prediction of both the infection and disease.

### Regional differences in sexual behaviour and HPV prevalence

Although the incidence of OPC in South America is similar to that in Western Europe and North America, the prevalence of HPV16 is reportedly low [65]. Latin America has an estimated overall HPV-positive head and neck cancer prevalence of between 3 and 4%, compared with 25% in European and North American populations [65–67]. This could partly be explained by differences in data collection and methods used to detect HPV. Despite Latin American countries having an average age of sexual debut between 18 and 19 years old [68], the International Head and Neck Cancer Epidemiology (INHANCE) Consortium found that these countries reported higher mean numbers of sexual partners (e.g. Brazil *n* = 22), compared with North American (e.g. USA, Atlanta *n* = 10) or European (e.g. Warsaw *n* = 15) populations [15]. Stratifying by region in our univariable MR analysis, we found a consistent protective effect for AFS and similarly, a consistent increased risk effect for NSP across all three regions (Europe, North America and South America), with evidence for the most precise effects in the North American population. In the largest pooled analysis, authors also report possible recall or reporting biases, given that some of the sexual behaviour interviews were carried out with family members nearby, in addition to small sample sizes (< 150 cases) [15] which may have affected their results.

### Confounding by other risk factors

While transmission of HPV to the upper aerodigestive tract is thought to be through oral sexual contact [9, 15–20], a more recent meta-analysis reported no association between oral sex practices and head and neck cancer risk [22]. This could be explained by the inclusion of older studies [22], which may not have captured the more recent rise in number of HPV-positive OPC cases which has been described by some as an ‘epidemic’ and predicted to overtake oral cancer within the next decade [69]. However, a study in the UK found that there was no change in the proportion of HPV-attributable cases from 2002 to 2011, although the incidence of OPC doubled over the same time period and national surveys have not described an increase in oral sex behaviour [1, 70]. In one multi-national study of 1626 men aged 18–73 years with 4-year follow-up, no association was detected between oral sexual behaviours and incident HPV infection, but oral oncogenic HPV was found to be more prevalent in current smokers compared with non-smokers [71]. Furthermore, tobacco exposure induces proinflammatory and immunosuppressive effects, which could potentially increase the likelihood of HPV infection and persistence [72, 73]. Since risk factors such as smoking and alcohol consumption are strongly associated with sexual behaviour and are well established in the aetiology of HNSCC, this may confound the relationship between sexual behaviours with HPV transmission and similarly OPC in observational studies [74, 75].

Although Mendelian randomization analysis minimizes the likelihood of confounding, since germline genetic variants should not theoretically be influenced by subsequent environmental confounders, pleiotropy is a major concern whereby genetic variants associated with the exposure (sexual behaviours- AFS and NSP) are related to the outcome (OPC) through alternative, independent biological pathways. We used a series of analyses to evaluate the potential for pleiotropy. We first performed several methods (MR-Egger [42], weighted median [43] and weighted mode [44]) which allow for the existence of horizontal pleiotropy and correct for this. We also identified and corrected for outlier SNPs most likely to exhibit pleiotropic effects. In univariable MR analyses, estimates were consistent with an effect of AFS and NSP on OPC risk. However, in further MR analysis taking lung cancer as a negative control, we observed the same direction of effect for AFS and NSP which we did not expect, since there is no plausible biological mechanism directly linking sexual behaviour with lung cancer risk. Evidence of an effect here indicates potential violation of the MR assumptions.

Strong genetic correlation between sexual behaviours and other risk factors such as smoking, alcohol and risk tolerance were found using LD score regression. The genetic instruments used in MR may therefore comprise variants which primarily influence other risk factors, which could induce correlated pleiotropy (Fig. 1). We conducted two subsequent analyses to evaluate this. The CAUSE approach provided limited evidence for any effect of NSP on OPC and was unable to distinguish an effect of AFS from the situation of correlated pleiotropy. We also performed multivariable MR to control for alcohol, smoking, risk tolerance and educational attainment, so as to determine the direct causal effect of sexual behaviours on OPC. Effect estimates attenuated when alcohol and smoking were taken into account in the multivariable MR models, again highlighting the role of potential pleiotropy in the genetic instruments for sexual behaviour.

### Strengths and limitations

MR was employed in this study in an attempt to overcome the drawbacks of conventional epidemiological studies. However, MR makes various assumptions which if violated may generate spurious conclusions. For example, sexual behaviours are difficult to instrument genetically due to measurement error (e.g. as a result of reporting bias) and because they are time-varying as well as context and culture-dependent. This could hamper the detection of genetic associations related to these traits which has implications for genetic instrument strength (the first assumption of MR), given the low percentage of variation explained (*R*^2^), as well as potential violation of the no measurement error (NOME) assumption, with relatively low *I*^2^ values. Similarly, it can be difficult to interpret genetic associations using educational attainment, when there is potential confounding by social and environmental factors, dynastic effects and assortative mating [76]. Therefore, MR estimates conditioning on educational attainment should be interpreted with caution. Causal estimates, particularly in multivariable MR, are subject to low power and hence wide confidence intervals. Therefore, we cannot discount the possibility of a small effect of sexual behaviour on OPC which might be consistent with the observational literature.

Additionally, the available genetic instruments are not specifically for oral sex, which is the conceptually relevant exposure and mode of HPV transmission. However, other sexual behaviours are likely to be correlated and developing genetic instruments for specific sexual activities pose some methodological and ethical challenges. While the random inheritance of genetic variants from parents to offspring means genotypes are typically much less associated with many potential confounders than directly measured exposures (the second MR assumption), a violation of this is created due to population stratification which can introduce confounding of genotype-outcome associations. Although the GWAS for both NSP and AFS were adjusted for genetic principal components, given that sexual behaviours are strongly socially patterned, residual population structure may reintroduce confounding into MR analysis. Although a rare outcome, there is potential sample overlap present as head and neck cancer cases were not excluded from previously published AFS or NSP GWAS; however, recent studies suggest the incurred bias is much less substantial than that due to weak instruments, or overestimation of the SNP-trait effect [77, 78]. Given some conditional F-statistics used in the multivariable MR were < 10, weak instrument bias is a possibility in these instances. This could result in difficulty interpreting our findings, particularly whether or not the observed attenuation in multivariable MR is statistically meaningful. Furthermore, for all the HPV GWAS, the mean chi-square from the LD score regression was small (< 1.1), indicating a lack of polygenic signal. This means that the results of both LD score regression and Mendelian randomization on HPV outcomes may not be informative.

The third major assumption of MR is the exclusion restriction principle (i.e. that the genetic variant affects the outcome exclusively through its effect on the exposure). We performed a series of comprehensive sensitivity analyses to evaluate potential violation of this assumption. While several pleiotropy-robust (MR-Egger, weighted median and weighted mode) and outlier exclusion methods provided limited evidence for violation of this assumption, the results of the lung cancer negative control analysis, CAUSE method and multivariable MR all suggested violation of the exclusion restriction assumption in the univariable MR of sexual behaviours on OPC risk. When multiple sources of evidence provide conflicting estimates, it is necessary to appraise the relative biases of the approaches in order to best “triangulate” evidence [79, 80]. In this instance, it is possible that the primary phenotype for the genetic variants used to instrument the sexual behaviours has been mis-specified. For example, the genetic variants may be primarily associated with other traits (e.g. risk taking) and indirectly to sexual behaviours via the primary traits. Similarly, sexual behaviour instruments may be associated with traits which do not have a direct negative connotation. In this instance, the Instrument Strength independent of Direct Effect (InSIDE) assumption of approaches such as MR-Egger is likely to be violated, whereas the CAUSE is less vulnerable to environmental confounders that are correlated with genetic variants than the other pleiotropy-robust methods.

Multivariable MR was also used to directly model the potential indirect effects of the genetic variants via other traits (smoking, alcohol, risk tolerance and educational attainment) and supported the conclusions of the CAUSE method. Finally, we could not distinguish between HPV-positive and HPV-negative oropharyngeal tumours in the GAME-ON summary data, which would require further analysis at an independent level or a GWAS of OPC stratified by HPV status. The GWAS-by-subtraction approach [81] could be useful to account for latent factors of other behavioural traits to identify more specific genetic instruments for sexual behaviour, if valid instruments for these traits exist. More serological data may become available in the UK Biobank and other clinical genetic studies, which could enhance power to evaluate potential the extent to which any effect of sexual behaviour on cancer risk is mediated by HPV.

## Conclusions

In conclusion, this study used a comprehensive series of MR analyses to investigate sexual behaviours in relation to OPC. We initially observed an association between genetically predicted AFS and NSP and risk of OPC using univariable MR. Despite using genetic variants strongly related to these traits in large-scale GWAS, further multivariate methods indicated violation of the core MR assumptions, likely due to correlated pleiotropy. There was evidence of some attenuation when alcohol and smoking were taken into account in the multivariable MR models, highlighting the importance of performing these further analyses, particularly when using genetic instruments which proxy complex behavioural traits.

## Supplementary Information

**Additional file 1.** Single nucleotide polymorphisms robustly and independently associated with age at first sex, number of sexual partners, risk tolerance, comprehensive smoking index, smoking initiation, drinks per week and educational attainment (years of schooling).

**Additional file 2: Supplementary information and Supplementary Tables and Figures.** **Table S1.** Univariable Mendelian randomization results for age at first sex and number of sexual partners on risk of oropharyngeal cancer using a more stringent r^2^ < 0.001. Abbreviations: AFS, age at first sex; NSP, number of sexual partners; IVW, inverse variance weighted; SE, standard error; OR, odds ratio; CI, confidence intervals; SNPs, single nucleotide polymorphisms. NSP OR represents the exponential change in odds of oropharyngeal squamous cell carcinoma per SD increase (0.94) in number of sexual partners. AFS OR represents the exponential change in odds of oropharyngeal squamous cell carcinoma per SD change (7.3-month delay) in age at first sex. **Table S2.** Univariable Mendelian randomization results of age at first sex with HPV seropositivity including sensitivity analyses. Abbreviations: IVW, inverse variance weighted; SE, standard error; OR, odds ratio; CI, confidence intervals; SNPs, single nucleotide polymorphisms. OR represents the exponential change in odds of HPV seropositivity per SD change (7.3-month delay) in age at first sex. GWAS were run for four HPV markers derived from UK Biobank, with HPV16 seropositivity described: if antigen L1 > 175; if antigen E6 > 120 or antigen E7 > 150. **Table S3.** Mendelian randomization results of number of sexual partners with HPV seropositivity including sensitivity analyses. Abbreviations: IVW, inverse variance weighted; SE, standard error; OR, odds ratio; CI, confidence intervals; SNPs, single nucleotide polymorphisms. OR represents the exponential change in odds of HPV seropositivity per SD increase (0.94) in number of sexual partners. GWAS were run for four HPV markers derived from UK Biobank, with HPV16 seropositivity described: if antigen L1 > 175; if antigen E6 > 120 or antigen E7 > 150. **Table S4.** Assessing weak instrument bias (F-statistic) and proportion of variance in the phenotype (*R*^2^) explained by age at first sex and number of sexual partners genetic instruments. Abbreviations: AFS, age at first sex; NSP, number of sexual partners. **Table S5.** Assessing heterogeneity and directional pleiotropy of single nucleotide polymorphism effect estimates for age at first sex and number of sexual partners on oropharyngeal cancer risk. Abbreviations: AFS, age at first sex; NSP, number of sexual partners; Q, Cochran’s Q-statistic; df, degrees of freedom; SE, standard error; P, p-value. **Table S6.** MR-PRESSO outliers detected results for age at first sex and number of sexual partners instruments on oropharyngeal cancer risk. Abbreviations: AFS, age at first sex; NSP, number of sexual partners; Q-stat, Cochran’s Q statistic. **Table S7.** MR-PRESSO results for age at first sex and number of sexual partners instruments on oropharyngeal cancer risk. Abbreviations: AFS, age at first sex; NSP, number of sexual partners; RSSobs, residual sum of squares observations. **Table S8.** Outlier corrected results for age at first sex and number of sexual partners instruments on combined oropharyngeal cancer. Abbreviations: IVW, inverse variance weighted; OR, odds ratio; CI, confidence intervals; SNPs, single nucleotide polymorphisms. NSP OR represents the exponential change in odds of oropharyngeal squamous cell carcinoma per SD increase (0.94) in number of sexual partners. AFS OR represents the exponential change in odds of oropharyngeal squamous cell carcinoma per SD change (7.3-month delay) in age at first sex. **Table S9.** SIMEX correction MR-Egger regression results for age at first sex and number of sexual partners instruments on oropharyngeal cancer risk (where I^2^ < 0.90). Abbreviations: AFS, age at first sex; NSP, number of sexual partners; I^2^, I-squared statistic; OR, odds ratio; CI, confidence intervals; *P*, p-value. **Table S10.** Univariable Mendelian randomization examining effects of age at first sex on positive and negative controls. Abbreviations: SE, standard error; OR, odds ratio; P, *p*-value; CI, confidence intervals; AFS, age at first sex. AFS OR represents the exponential change in odds of cervical or lung cancer per SD change (7.3-month delay) in age at first sex. **Table S11.** Univariable Mendelian randomization examining effects of number of sexual partners on positive and negative controls. Abbreviations: SE, standard error; OR, odds ratio; P, *p*-value; CI, confidence intervals; NSP, number of sexual partners; NSP OR represents the exponential change in odds of cervical or lung cancer per SD increase (0.94) in number of sexual partners. **Table S12.** Assessing directional pleiotropy through MR-Egger intercept for univariable MR positive and negative control analyses. Abbreviations: AFS, age at first sex; NSP, number of sexual partners; SE, standard error; P, p-value. **Table S13.** Assessing heterogeneity of single nucleotide polymorphism effect estimates in inverse variance weighted and MR-Egger regression for univariable MR positive and negative control analyses. Abbreviations: AFS, age at first sex; NSP, number of sexual partners; Q, Cochran’s Q-statistic; IVW, inverse variance weighted; df, degrees of freedom; P, p-value. **Table S14.** MR-PRESSO outliers detected results for age at first sex and number of sexual partners instruments on positive and negative controls. Abbreviations: AFS, age at first sex; NSP, number of sexual partners; Q-stat, Cochran’s Q statistic. **Table S15.** Outlier corrected results for age at first sex and number of sexual partners instruments on positive and negative controls. Abbreviations: SE, standard error; OR, odds ratio; P, *p*-value; CI, confidence intervals; AFS, age at first sex; NSP, number of sexual partners. AFS OR represents the exponential change in odds of cervical or lung cancer per SD change (7.3-month delay) in age at first sex. NSP OR represents the exponential change in odds cervical or lung cancer per SD increase (0.94) in number of sexual partners. **Table S16.** Causal Analysis Using Summary Effect estimates (CAUSE) results for age at first sex on risk of oropharyngeal cancer. Abbreviations: OPC, oropharyngeal cancer; ELPD, expected log pointwise posterior density; se, standard error; *γ* (gamma), estimate of causal effect if causal model is correct; η (eta), estimate of correlated pleiotropy; q, proportion of effect due to correlated pleiotropy; CI, confidence intervals; NA, non-applicable. **Table S17.** Causal Analysis Using Summary Effect estimates (CAUSE) results for number of sexual partners on risk of oropharyngeal cancer. Abbreviations: OPC, oropharyngeal cancer; ELPD, expected log pointwise posterior density; se, standard error; *γ* (gamma), estimate of causal effect if causal model is correct; η (eta), estimate of correlated pleiotropy; q, proportion of effect due to correlated pleiotropy; CI, confidence intervals; NA, non-applicable. **Table S18.** Overlapping single nucleotide polymorphisms identified between genetic instruments used in multivariable Mendelian randomization. Abbreviations: AFS, age at first sex; NSP, number of sexual partners; RT, risk tolerance; CSI, comprehensive smoking index; SI, smoking initiation; DPW, drinks per week. **Table S19.** LD Score Regression results for all exposures. Abbreviations: AFS, age at first sex; NSP, number of sexual partners; CSI, comprehensive smoking index; SI, smoking initiation; DPW, drinks per week; RT, risk tolerance; rg, genetic correlation; SE, bootstrap standard error of genetic correlation, h^2^ obs = estimated SNP heritability of the second exposure , h^2^ obs se = bootstrap standard error of the SNP heritability estimate, h^2^ int = LD score regression intercept for the second exposure, h^2^ int se = bootstrap standard error of the intercept, gcov int = estimated genetic covariance between exposure 1 and 2, gcov int se = bootstrap standard error of the genetic covariance. **Table S20.** Assessing directional pleiotropy through MR-Egger intercept for multivariable MR analysis on oropharyngeal cancer. Abbreviations: AFS, age at first sex; NSP, number of sexual partners; SE, standard error; P, p-value; CSI, comprehensive smoking index; SI, smoking initiation; DPW, drinks per week; RT, risk tolerance. **Table S21.** Bidirectional Mendelian randomization analysis for age at first sex on other risk factors. Abbreviations: AFS, age at first sex; SNP, single nucleotide polymorphism; SE, standard error; CSI, comprehensive smoking index; SI, smoking initiation; DPW, drinks per week; RT, risk tolerance; EA, educational attainment. **Table S22.** Bidirectional Mendelian randomization analysis for number of sexual partners on other risk factors. Abbreviations: NSP, number of sexual partners; SNP, single nucleotide polymorphism; SE, standard error; CSI, comprehensive smoking index; SI, smoking initiation; DPW, drinks per week; RT, risk tolerance; EA, educational attainment. **Table S23.** Multivariable Mendelian randomization for age at first sex and number of sexual partners with risk lung cancer. Abbreviations: IVW, inverse variance weighted; OR, odds ratio; CI, confidence intervals; *P, p*-value; Q-stat, Cochran’s Q statistic; F-stat, conditional F-statistic. AFS OR represents the exponential change in odds of oropharyngeal squamous cell carcinoma per SD change (7.3-month delay) in age at first sex. NSP OR represents the exponential change in odds of oropharyngeal squamous cell carcinoma per SD increase (0.94) in number of sexual partners. **Table S24.** Assessing directional pleiotropy through MR-Egger intercept for multivariable MR analysis on lung and cervical cancer. Abbreviations: AFS, age at first sex; NSP, number of sexual partners; SE, standard error; P, *p*-value; CSI, comprehensive smoking index; SI, smoking initiation; DPW, drinks per week; RT, risk tolerance. **Figure S1** Forest plots showing Mendelian randomization results for age at first sex and number of sexual partners single nucleotide polymorphisms with risk of oropharyngeal cancer in GAME-ON. Effect estimates are reported on the log odds scale with 95% confidence intervals. **A.** Age at first sex point estimate represents the exponential change in odds oropharyngeal squamous cell carcinoma per SD change (7.3 month delay) in age at first sex. **B.** Number of sexual partners point estimate represents the exponential change in odds of oropharyngeal squamous cell carcinoma per SD increase (0.94) in number of sexual partners. **Figure S2** Scatter plots for age at first sex and number of sexual partners single nucleotide polymorphisms effect on oropharyngeal cancer in GAME-ON. Scatter plots for **A.** age at first sex and **B.** number of sexual partners single nucleotide polymorphisms effect on oropharyngeal cancer in GAME-ON. Coloured lines indicating Mendelian Randomization test as described in the key above. **Figure S3** Leave one out plots for age at first sex and number of sexual partners single nucleotide polymorphisms effect on oropharyngeal cancer in GAME-ON. Leave one out plots for **A.** age at first sex and **B.** number of sexual partners. **Figure S4** Scatter and leave one out plots for age at first sex and number of sexual partners single nucleotide polymorphisms effect on risk of cervical cancer. Scatter and leave one out plots for **A.** age at first sex and **B.** number of sexual partners single nucleotide polymorphisms effect on cervical cancer. Coloured lines indicating Mendelian Randomization test as described in the key above. **Figure S5** Scatter and leave one out plots for age at first sex and number of sexual partners single nucleotide polymorphisms effect on risk of C. trachomatis seropositivity. Scatter and leave one out plots for **A.** age at first sex and **B.** number of sexual partners single nucleotide polymorphisms effect on Chlamydia trachomatis seropositivity. Coloured lines indicating Mendelian Randomization test as described in the key above. **Figure S6** Scatter and leave one out plots for age at first sex and number of sexual partners single nucleotide polymorphisms effect on risk of lung cancer. Scatter and leave one out plots for **A.** age at first sex and **B.** number of sexual partners single nucleotide polymorphisms effect on lung cancer. Coloured lines indicating Mendelian Randomization test as described in the key above. **Figure S7** Scatter and leave one out plots for age at first sex and number of sexual partners single nucleotide polymorphisms effect on risk of oral cancer. Scatter and leave one out plots for **A.** age at first sex and **B.** number of sexual partners single nucleotide polymorphisms effect on oral cancer. Coloured lines indicating Mendelian Randomization test as described in the key above. **Figure S8** Causal Analysis Using Summary Effect estimates (CAUSE) results for age at first sex on oropharyngeal cancer. Plots showing sharing, causal and expected log pointwise posterior density (ELPD) models for age at first sex on oropharyngeal cancer. Causal Analysis Using Summary Effect estimates (CAUSE) suggests there is relatively similar evidence for sharing (correlated pleiotropy) and causal models compared to the null (no effect) model. Comparing both shared and causal models, there is limited evidence that the causal model fits the data better than the sharing model, indicating that correlated pleiotropy could not be discounted. Gamma, estimate of causal effect if causal model is correct; Eta estimate of correlated pleiotropy. **Figure S9** Causal Analysis Using Summary Effect estimates (CAUSE) results for number of sexual partners on oropharyngeal cancer. Plots showing sharing, causal and expected log pointwise posterior density (ELPD) models for number of sexual partners on oropharyngeal cancer. Neither shared nor causal models appear to fit in comparison to the null model, providing limited evidence for a causal effect of number of sexual partners on oropharyngeal cancer risk. Gamma, estimate of causal effect if causal model is correct; Eta estimate of correlated pleiotropy. **Figure S10** Heat map of LD Score regression results for all exposures. Abbreviations: AFS, age at first sex; NSP, number of sexual partners; CSI, comprehensive smoking index; SI, smoking initiation; DPW, drinks per week; EA, educational attainment; RT, risk tolerance. **Figure S11** Heat map of LD Score regression results for all number of sexual partners and age at first sex on HPV-seropositivity. Abbreviations: AFS, age at first sex; NSP, number of sexual partners; HPV, human papilloma virus. **Figure S12** Forest plot showing multivariable Mendelian randomization results for age at first sex and number of sexual partners single nucleotide polymorphisms with risk of lung cancer. Effect estimates on oropharyngeal cancer risk are reported on the log odds scale with 95% confidence intervals. UVMR, univariable Mendelian randomization; MVMR, multivariable Mendelian randomization. **A.** Age at first sex OR represents the change in odds of lung cancer per SD change (7.3-month delay) in age at first sex. **B.** Number of sexual partners OR represents the change in odds of lung cancer per SD change (0.94) in number of sexual partners. Comprehensive smoking index (dark orange), smoking initiation (teal blue), alcoholic drinks per week (yellow), risk tolerance (green), educational attainment (light orange). The MVMR effect is the MR effect after accounting for this variable.

## Data Availability

Summary-level analysis was conducted using publicly available GWAS data. Full summary statistics for the GAME-ON outcome data GWAS can be accessed via dbGAP (OncoArray: Oral and Pharynx Cancer; study accession number: phs001202.v1.p1, August 2017 at: https://www.ncbi.nlm.nih.gov/projects/gap/cgi-bin/study.cgi?study_id=phs001202.v1.p1) [82]. There is one selected publication by Lesseur et al. related to this data [31]. Lung cancer GWAS data is available via dbGAP (Transdisciplinary Research Into Cancer of the Lung (TRICL) - Meta Analysis; dbGaP study accession number: phs000877.v1.p1, March 2015 at: https://www.ncbi.nlm.nih.gov/projects/gap/cgi-bin/study.cgi?study_id=phs000877.v1.p1) [83], with three selected publications relevant to this study [48, 84, 85]. Summary-level data for the main exposures used in this study were derived from the relevant publications for age at first sex [28] and number of sexual partners [26], smoking initiation, 60 SNPs for alcoholic drinks per week [51], comprehensive smoking index [53], 123 for risk tolerance [26], and 317 SNPs for educational attainment [52]. Cervical cancer, HPV and *C. trachomatis* GWAS data were all derived using UK Biobank as described. Access to UK Biobank (https://www.ukbiobank.ac.uk/) data is available to researchers through application and is described in the relevant publication by Bycroft et al. [30]. UK Biobank approval was given for this project (ID 40644 “Investigating aetiology, associations and causality in diseases of the head and neck”) and UK Biobank GWAS data was also accessed under the application (ID 15825 “MR-Base: an online resource for Mendelian randomization using summary data”- Dr Philip Haycock).” Genetic instruments derived from UK Biobank may also be available via the IEU OpenGWAS project (https://gwas.mrcieu.ac.uk/) with relevant publications to support this resource from Elsworth et al. [86] and Hemani et al. [87]. For the purpose of open access, the authors have applied a CC BY public copyright licence to any Author Accepted Manuscript version arising from this submission. MR analyses were conducted using the “TwoSampleMR” package in R (version 3.5.3). A copy of the code and all files used in this analysis is available at GitHub [88] via https://github.com/rcrichmond/sexual_behaviours_opc.

## Abbreviations

AFS: Age at first sex
CAUSE: Causal Analysis Using Summary Effect estimates
CIL: Lower confidence interval
CIU: Upper confidence interval
GAME-ON: Genetic Associations and Mechanisms in Oncology Network
GWAS: Genome-wide association study
HPV: Human papilloma virus MR: Mendelian randomization
MVMR: Multivariable Mendelian randomization
NSP: Number of sexual partners
OR: Odds ratio
*P*: *p* value
SD: Standard deviation
SE: Standard error
